# Efficacy and suitable indication of colorectal endoscopic submucosal dissection using a balloon‐assisted endoscope

**DOI:** 10.1002/jgh3.12247

**Published:** 2019-08-19

**Authors:** Yuichiro Kuroki, Kunio Asonuma, Natsumi Uehara, Toshiyuki Endo, Reika Suzuki, Yorimasa Yamamoto, Masatsugu Nagahama

**Affiliations:** ^1^ Department of Gastroenterology Showa University Fujigaoka Hospital Kanagawa Japan

**Keywords:** colonoscopy, colorectal tumor, endoscopic submucosal dissection, single‐balloon endoscopy

## Abstract

**Background and Aim:**

Cases of colorectal endoscopic submucosal dissection (ESD) with poor maneuverability are often encountered. We aimed to evaluate the efficacy of balloon‐assisted endoscopy (BAE) for such cases.

**Methods:**

We confirmed maneuverability preoperatively in 400 consecutive cases of colorectal ESD performed at a single center from April 2011 to April 2018. A total of 83 deep colon cases judged as having poor maneuverability were retrospectively reviewed; 54 cases underwent BAE with a single balloon endoscope (group B), and 29 cases underwent conventional procedures without BAE (group C). Tumor size, procedure duration, dissection speed, en bloc resection rate, histology, and associated complications were compared between groups.

**Results:**

The mean tumor size, tumor invasiveness, fibrosis, and complications did not differ between groups. Although the en bloc resection rate did not differ (both 98%), the groups significantly differed with regard to the R0 resection rate (B: 96%; C: 83%; *P* = 0.048). Overall, the procedure duration (B: 51 min; C: 70 min; *P* = 0.17) and dissection speed (B: 19.4 mm^2^/min; C: 17.4 mm^2^/min; *P* = 0.13) were not significantly different between groups. However, the dissection speed for lesions in the cecum/ascending colon was significantly faster in group B than in group C (B: 22.3 mm^2^/min; C: 11.3 mm^2^/min; *P* = 0.037).

**Conclusions:**

In cases of colorectal ESD with poor maneuverability, the use of BAE contributed to an improvement in the R0 resection rate. In addition, BAE contributed to a quicker dissection speed for lesions located in the cecum/ascending colon.

## Graphical abstract

In cases of colorectal endoscopic submucosal dissection with poor maneuverability, the use of balloon‐assisted endoscopy (BAE) contributed to improvement in the R0 resection rate. In addition, BAE contributed to quicker dissection speed for lesions located in the cecum/ascending colon. This is an important point for deciding which to select, BAE or conventional endoscopy.

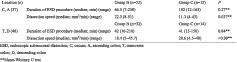

## Introduction

Endoscopic submucosal dissection (ESD) has gradually emerged as a feasible treatment option for colorectal tumors with the development of improved techniques and specialized devices.^1^, ^2^, ^3^ Although colorectal ESD is recognized as a standard technique for colorectal tumors, difficult cases are often encountered. Factors creating difficulties for ESD include fibrosis of the submucosal layer, fold convergence, lesion type, colon location, and poor endoscopic maneuverability.^4^, ^5^, ^6^, ^7^ Among these factors, poor endoscopic maneuverability can be relatively stressful for colorectal ESD.

Various efforts have been made to improve difficult conditions. As a countermeasure, body position and endoscope can be changed appropriately. In addition, there are several recent reports^8^, ^9^, ^10^, ^11^ on the usefulness of colorectal ESD with modified balloon‐assisted endoscopy (BAE).^12^ In the present study, we examined the efficacy of colorectal ESD using a balloon‐assisted endoscope compared to that with a conventional scope in cases judged as having poor operability on preoperative colonoscopy. In addition, we examined the efficacy according to colonic location.

## Methods

### *Patients*

**Figure 1:**
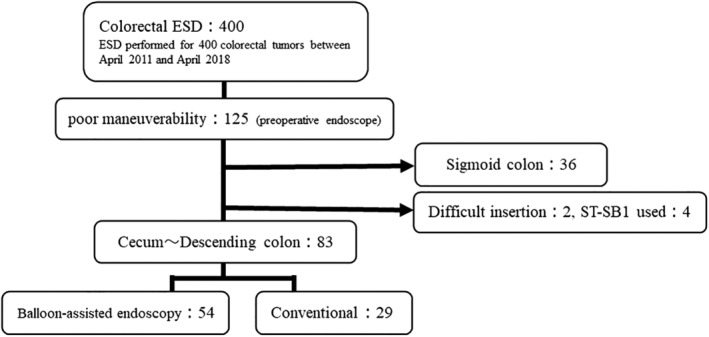
Study flow diagram. ESD, endoscopic submucosal dissection; ST‐SB1, single‐balloon overtube for the small intestine (Olympus).

Patients treated between April 2011 and April 2018 at our hospital were retrospectively reviewed. Maneuverability and indication were confirmed on preoperative endoscopy (CF‐HQ290ZI or CF‐H260AZI; Olympus, Tokyo, Japan), with magnifying function, in 400 consecutive cases of colorectal ESD performed during the study period. Poor maneuverability comprised paradoxical movement of the endoscope,^10^ poor control with adhesion after abdominal surgery, and redundant colon. Of the 125 cases judged as having poor maneuverability, 36 cases of sigmoid colon without the use of BAE, 2 cases of incomplete total colonoscopy, and 4 cases with the use of a single balloon overtube for the small intestine (ST‐SB 1; Olympus) were excluded. Finally, 83 deep colon cases judged as having poor maneuverability were included. Among these, 54 cases underwent BAE, introduced in March 2015 at our hospital (group B), and 29 cases underwent conventional procedures before BAE was introduced (group C) (Fig. 1). This study was approved by our Institutional Review Board (No. F2018C12); all patients were provided with the opportunity to opt out of the study.

### *Endoscopic system*

**Figure 2:**
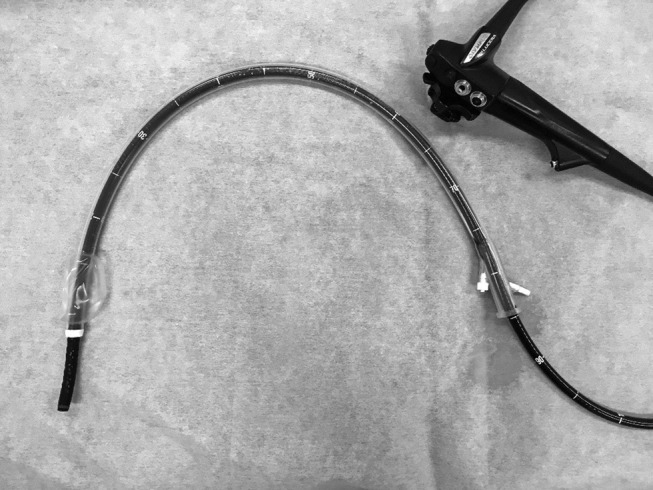
Single‐balloon endoscopy (Olympus).

A water jet system‐furnished ultra‐slim endoscope (PCF‐Q260J; Olympus) was used. When scope operability was poor because of paradoxical movement or adhesion, a balloon‐assisted endoscope with a hydrophilic‐coated silicone splinting tube (ST‐CB 1; Olympus) was used (Fig. 2). A transparent disposable attachment (D‐201‐11 804; Olympus) or a short‐type small‐caliber‐tip transparent hood (Fujifilm; Tokyo, Japan) was placed on the endoscopic tip. For all cases, carbon dioxide was used for insufflation. The electrosurgical unit was an ESG‐100 (Olympus). We primarily used a dual knife (KD650Q; Olympus); however, a hook knife (KD‐260R; Olympus) was added in cases with severe fibrosis and a vertical approach.

### *ESD procedure*

ESD was usually performed under conscious sedation using midazolam (1–3 mg/body), pethidine hydrochloride (35 mg/body), or both. At the time of all ESD, we first intubated the scope to the cecum and cleaned the bowel with a water jet. All ESD procedures were performed as described previously.^13^, ^14^ A submucosal injection of a hyaluronic acid solution mixed with a small amount of indigo carmine and 0.1% epinephrine was administered. Marking of the incision was usually unnecessary as the border between the tumor and normal tissue becomes quite clear in colorectal tumors with the application of indigo carmine. After adequate submucosal injection, the knife was gently applied on the incision line, and an incision was made.

Dissection was performed using the “forced coagulation” mode (30 W) of the ESG‐100. First, we dissected into the inferior of the tumor. Once adequate space for the tip hood was created under the tumor, the submucosal layer under the tumor could be directly observed. Furthermore, appropriate traction was created by the tip hood under the tumor, increasing the effectiveness of the dissection. An appropriate change in positioning evaginated the dissected tumor, increasing dissection efficiency. Fibrotic tissues required careful dissection.

### *Measured characteristics and outcomes*

Tumor size and location, resected specimen size, duration of cecum intubation, procedure duration, dissection speed, degree of fibrosis (F0–F2),^15^ en bloc resection rate, R0 resection (defined as en bloc resection with free vertical and horizontal margins) rate, histology, and associated complications were compared between groups. Procedure duration and dissection speed were further analyzed according to the colonic location of the lesion (cecum/ascending colon; transverse/descending colon).

### *Statistical analysis*

All variables are reported as medians. Group differences were evaluated using the Mann–Whitney *U* test for continuous variables and the χ^2^ test for dichotomous variables. A *P* < 0.05 was considered statistically significant. All statistical analyses were performed using EZR (Saitama Medical Center, Jichi Medical University, Saitama, Japan), which is a graphical user interface for R (The R Foundation for Statistical Computing, Vienna, Austria). More precisely, it is a modified version of the R‐commander, designed to add statistical functions frequently used in biostatistics.^16^

## Results

### *Patient characteristics*

Patient characteristics are presented in Table 1. There were no significant group differences in gender, tumor location, macroscopic type, or operator (expert: colorectal ESD experience >100 cases). However, group B tended to be slightly older than group C (72 (48–84) *vs* 68 (41–87), *P* = 0.07). Past abdominal surgery did not significantly differ between the groups (13/54 (24.1%) *vs* 8/29 (27.6%), *P* = 0.79).

**Table 1:** Clinical characteristics of patients in the balloon‐assisted endoscope (group B) and conventional endoscope (group C) groups

	Group B	Group C	*P*
Number of lesions	54	29	
Gender (male/female)	37/17	22/7	0.61†
Age (years), median (range)	72 (48–84)	68 (41–87)	0.07‡
Location (C, A/T, D)	22/32	15/14	0.36†
Macroscopic type (LST‐G/LST‐NG/others)	22/28/4	8/19/2	0.48†
Operator (expert/trainee)	40/14	21/8	>0.99†
Previous abdominal surgery	13 (24.1%)	8 (27.6%)	0.79†

† Chi‐squared test.

‡ Mann–Whitney *U* test.

Expert, colorectal endoscopic submucosal dissection experience >100 cases.

A, ascending colon; C, cecum; D, descending colon; D, descending colon; LST‐G, laterally spreading tumor (granular type); LST‐NG, laterally spreading tumor (non‐granular type); Others, tumors including submucosal tumor and protruded type; T, transverse colon.

### *Clinical outcomes and courses*

Clinical outcomes are summarized in Table 2. Median ESD procedure duration was not significantly different between the groups; however, it tended to be slightly shorter in group B than in group C (B: 51 min; C: 70 min; *P* = 0.17). Similarly, median ESD dissection speed was not significantly different between the groups; however, it tended to be slightly faster in group B than in group C (B: 19.4 mm^2^/min; C: 17.4 mm^2^/min; *P* = 0.13). Duration of cecum intubation was significantly longer in group B than in group C (B: 9.5 min; C: 6 min; *P* < 0.001). The rate of en bloc resection, degree of fibrosis, and histological findings did not differ between groups (*P* > 0.99). However, the R0 resection rate was significantly higher in group B than in group C (52/54 (96%) *vs* 24/29/ (83%), *P* = 0.048). Two cases did not undergo en bloc resection, with one case in each group. The case in group B was a laterally spreading tumor (nodular‐mixed type) involving the ileocecal valve and measuring over 50 mm. ESD for the lesion was abandoned because the lesion had rich fat and severe vascular fibrosis. The case in group C was a sessile‐type polyp located at the ascending colon and measuring approximately 25 mm. It was divided into three parts, with a snare for the lesion because of intraoperative perforation. The histological evaluation demonstrated horizontal margin‐positive and vertical margin‐negative intramucosal cancer.

**Table 2:** Clinical outcomes in the balloon‐assisted endoscope (group B) and conventional endoscope (group C) groups

	Group B	Group C	*P*
Tumor size (median; mm) (range)	25 (10–80)	25 (10–80)	0.69†
Size of resected specimens (median; mm) (range)	32 (15–85)	35 (10–85)	0.68†
Duration of cecum intubation (median; min) (range)	9.5 (4–20)	6 (2–12)	<0.001†
Duration of ESD procedure (median; min) (range)	51 (7–250)	70 (12–165)	0.17†
Dissection speed (median; mm^2^/min) (range)	19.4 (5–52)	17.4 (4–43)	0.13†
Fibrosis (F0‐1/F2)	49/5	27/2	>0.99‡
Pathology, *n* (%)			0.74‡
Adenoma, Intramucosal cancer	46 (85.2)	26 (89.6)	
SM	8 (14.8)	3 (3.4)	
En bloc resection rate (%)	53/54 (98.1)	28/29 (96.6)	>0.99‡
R0 resection rate (%)	52/54 (96.3)	24/29 (82/8)	0.048‡
Perforation rate (%)	1/54 (1.9)	1/29 (3.4)	>0.99‡
Postoperative bleeding rate (%)	1/54 (1.9)	0/29 (0)	>0.99‡

† Mann–Whitney *U* test.

‡ Chi‐squared test.

ESD, endoscopic submucosal dissection; SM, submucosal; R0 resection, defined as en bloc resection with free vertical and horizontal margin.

The rates of intraoperative perforation in groups B and C were 1.9 and 3.4%, respectively, without a significant difference between the groups (*P* > 0.99). Similarly, the rates of postoperative bleeding in groups B and C were 1.9 and 0%, respectively, without a significant difference between the groups (*P* > 0.99). Two cases of perforation (one in each group) were conservatively managed by medical treatment after endoscopic closure using endoclips. A case of postoperative bleeding in group B was managed conservatively using endoclips. There were no complications related to the use of a balloon overtube.

ESD procedure duration and dissection speed were further analyzed according to location (from the cecum to ascending colon, from the transverse to descending colon) (Table 3). For tumors in the cecum to ascending colon, the ESD procedure duration did not significantly differ between the groups (66.5 *vs* 102 min, *P* = 0.27). However, ESD dissection speed was significantly faster in group B than in group C (22.3 *vs* 11.3 mm^2^/min, *P* = 0.037). For tumors in the transverse to descending colon, the ESD procedure duration and dissection speed did not significantly differ between the groups (B: 42 min; C: 41 min; *P* = 0.84 for procedure duration) (B: 18.4 mm^2^/min; C: 20.6 mm^2^/min; *P* > 0.99 for dissection speed).

**Table 3:** Comparisons between balloon‐assisted endoscope (group B) and conventional endoscope (group C) groups according to colonic location

Location (*n*)				*P*
		Group B (*n* = 22)	Group C (*n* = 15)	
C, A (37)	Duration of ESD procedure (median; min) (range)	66.5 (7–250)	102 (12–165)	0.27†
	Dissection speed (median; mm^2^/min) (range)	22.3 (8–51)	11.3 (4–43)	0.037†
		Group B (*n* = 32)	Group C (*n* = 14)	
T, D (46)	Duration of ESD procedure (median; min) (range)	42 (16–218)	41 (15–150)	0.84†
	Dissection speed (median; mm^2^/min) (range)	18.4 (5–45.7)	20.6 (4.5–40)	>0.99†

† Mann–Whitney *U* test.

A, ascending colon; C, cecum; D, descending colon; ESD, endoscopic submucosal dissection; T, transverse colon.

## Discussion

Difficult cases with poor endoscopic maneuverability are often encountered in colonic endoscopic resection. There are various causes of poor endoscopic maneuverability, such as paradoxical movement, postabdominal surgery adhesions, and redundant colon. BAE was originally developed for observation and treatment of the small intestine^17^ but has also been applied in cases of difficult colonoscope insertion.^18^, ^19^ BAE is also used for the treatment of colorectal polypectomies and in endoscopic mucosal resection (EMR).^20^

Ohya *et al*.^11^ first reported the efficacy of BAE for ESD cases with poor endoscopic operability using a single‐balloon system (ST‐SB 1; Fujifilm) for the small intestine. Subsequently, Asayama *et al*.^10^ reported that BAE using ST‐CB 1 improved difficulties due to paradoxical movement and poor control with adhesion in 20 consecutive deep colon ESDs, allowing high rates of en block and histological resections. Furthermore, Ohata *et al*.^9^ reported that ESD cases with severe adhesions could be resected using ST‐CB 1. In addition, Yamashita *et al*.^8^ reported the efficacy of balloon‐assisted ESD, comparing double balloon‐assisted ESD to nonballoon‐assisted ESD using propensity score matching.

Consistent with previous reports, the present study demonstrated the efficacy of colonic ESD using BAE, specifically in deep colon cases with poor maneuverability. Colorectal ESD using a balloon‐assisted endoscope contributed to a shorter resection time and improvement in the R0 resection rate. It is easy to understand that the procedural time will be shortened when endoscopic operability is stable. In addition, although the en bloc resection rate did not differ between groups, the R0 resection rate was improved, likely because an accurate resection was possible with more stable scope operability. However, many of the group C cohort were patients with ESD treated before the introduction of BAE in March 2015, while the group B cohort comprised patients with ESD treated after that the introduction of BAE. Thus, a learning curve effect, with improvements over time, may have contributed to an increase in dissection speed in this study. Furthermore, in the present study, as well as in previous reports, there were no complications caused by the balloon endoscope itself.

In addition, in the present study, ESD using a balloon‐assisted endoscope contributed to a shorter procedural time and quicker dissection speed for lesions located in the cecum/ascending colon. As the deeper colon (cecum and ascending colon) is fixed in the mesentery, we thought that if the distal colon was fixed in the balloon tube, the scope would be stable. On the other hand, the cecum intubation duration was significantly longer in group B than in group C, with a median difference of 3.5 min. This amount of time was trivial in terms of the duration of the total procedure and did not cause any problems. In a meta‐analysis, the cecum intubation duration using single‐balloon endoscopy was reported to be 19 min.^21^ Because the present study did not include cases of incomplete or previous difficult colonoscopies, the duration of cecum intubation may be shorter than that in previous reports. The present results suggest that BAE may be used positively in cases of poor endoscopic operability at the cecum and ascending colon. This is an important point in deciding which to select, BAE or conventional endoscopy. Furthermore, fogging of the endoscopic lens can easily occur in deep colon cases involving the cecum or ascending colon as these areas have rich fat in the colonic submucosa. In such cases, the scope can be withdrawn and inserted while holding the splinting tube in the gut and attaching the tip hood to the tip of the endoscope,^10^ making the cleaning of the scope lens easier.

**Figure 3:**
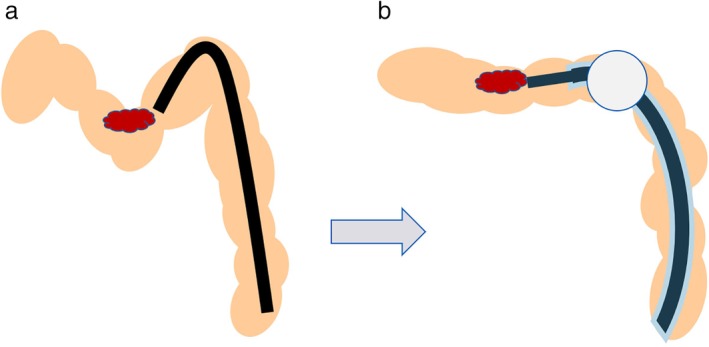
The colonic wall stretched by the balloon to the anus side. (a) The endoscope is in a vertical approach to the lesion, with a sharp bend in the mid‐transverse colon. (b) Using a balloon‐assisted endoscope provides an anchor for the colon wall, shortening and straightening it. As a result, it is possible to approach the target area with the endoscope parallel to the tumor.

**Figure 4:**
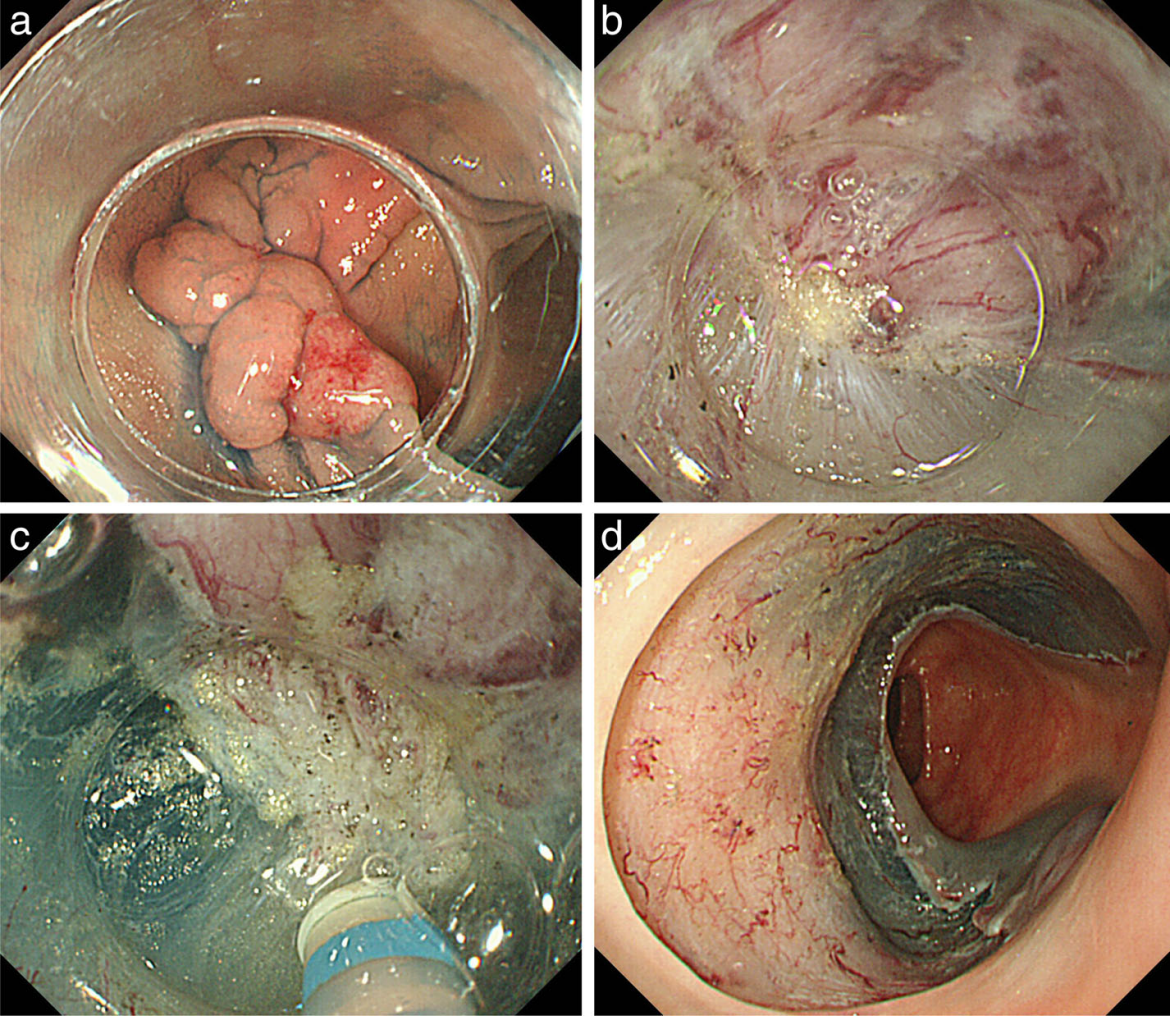
The tumor with fibrosis located in the mid‐transverse colon. (a) Using a balloon‐assisted endoscope, the approach is parallel to the lesion. (b, c) Submucosal dissection with fibrosis. In addition to providing good operability, balloon‐assisted endoscopy allowed the knife to be kept parallel to the muscular layer. (d) Ulcer bed after complete resection. Histological evaluation demonstrated margin‐negative intramucosal cancer.

In contrast to the results for the cecum/ascending colon, procedural time and dissection speed did not differ between the groups for lesions located in the transverse/descending colon. However, BAE appeared to be effective for some transverse colon cases. The central part of the transverse colon may exist as a sharp bend for patients with redundant and sagging transverse colons. As reported by Ohya *et al*.,^11^ using a balloon‐assisted endoscope (ST‐SB 1) provides an anchor for the colon wall, potentially shortening and straightening it. This effect was observed in the present study when using the ST‐CB 1. There were some cases in which the endoscope changed from a vertical to a parallel approach to the lesion (Figs 3 and 4). Thus, in addition to providing good operability in transverse colon cases, BAE may allow the knife to be kept parallel to the muscular layer. As group B was statistically not inferior to group C, the present results suggest that BAE may be used positively in cases in which keeping the knife parallel to the muscular layer is needed, even in the transverse/descending colon.

Despite the present positive results, we consider BAE unnecessary for cases with good endoscopic operability; the necessity and cost of BAE must be considered. Therefore, checking endoscopic operability by preoperative colonoscopy is important. Although such cases were excluded in the present study, Yamashita *et al*.^8^ reported that BAE is useful even in cases of previous incomplete colonoscopy. We agree that ESD with a BAE should be used for difficult colonoscopy insertions. In the future, a good strategy for colorectal ESD with poor maneuverability in the sigmoid colon should be considered.

The present study has some limitations. First, in addition to the small sample size, the study was conducted at a single institution, had a retrospective cohort design, and the cohorts differed in the duration of the procedure within the study period; there was no randomization. Second, the evaluation of scope operability was made subjectively during the preoperative colonoscopy; this is difficult to evaluate objectively.

In conclusion, in cases of colorectal ESD with poor maneuverability, BAE contributed to an improvement in the R0 resection rate. In addition, BAE contributed to a quicker dissection speed for lesions located in the cecum/ascending colon. Thus, BAE may contribute to the further spread of colorectal ESD.
